# Opioids and Sickle Cell Disease: From Opium to the Opioid Epidemic

**DOI:** 10.3390/jcm10030438

**Published:** 2021-01-23

**Authors:** Samir K. Ballas

**Affiliations:** Cardeza Foundation for Hematologic Research, Department of Medicine, Sidney Kimmel Medical College, Thomas Jefferson University, Philadelphia, PA 19107, USA; samir.ballas@jefferson.edu

**Keywords:** sickle cell disease, opium, opioids, epidemic, mortality, pandemic

## Abstract

Sickle cell disease (SCD) is an inherited disorder of hemoglobin structure. The clinical effects of the sickle gene are pleiotropic in nature causing multiple phenotypic expressions associated with the various complications of the disease. The hallmark of the disease is pain that could be acute, chronic, nociceptive, or neuropathic that could occur singly or in various combinations. The acute vaso-occlusive painful crisis (VOC) is the most common cause of admissions to the Emergency Department and/or the hospital. Although progress has been made in understanding the pathophysiology of SCD as well as in developing preventive and curative therapies, effective pain management continues to lag behind and depend mostly on the use of opioids. This review describes the history of opioids from the ancient times of opium to the current use of the many controversial opioids. In addition, the major cause of death of patients with SCD is the complications of the disease itself and not the use of opioids. The use of opioids by patients with SCD has been stable over the years. Judicious use of opioids to treat sickle cell pain according to available guidelines could minimize the unnecessary suffering experienced by patients with SCD.

## 1. Introduction

Sickle cell disease (SCD) is a complex genetic disorder caused by a missense mutation in the human β globin gene (HBB) leading to the sickle hemoglobin (Hb) variant HbS. Patients with SCD can be homozygous (HbSS) or heterozygous for the mutation (HbSC) [1]. HbS–β0-thalassemia is a form of SCD that is clinically similar in severity to HbSS, whereas HbS-β+-thalassemia is a milder form of SCD. Sickle cell disease predominantly affects individuals of African descent and is the most common hemoglobinopathy, with approximately 300,000 new cases each year and millions of patients affected globally. In the United States, there are more than 230,000 hospital admissions related to SCD annually at an economic cost of $2.4 billion [2]. Acute episodes of pain, also commonly referred to as vaso-occlusive crises (VOCs), are not only the primary presenting morbidity associated with SCD, but also the most common cause of admission to the Emergency Department or Hospital in approximately 95% of cases [3]. It is estimated that up to 100,000 patients in the USA have SCD [2].

The major objective of this paper is to describe what the opioids really are and how they came about over the years. Opium has been used in different countries and cultures for millennia, but only in the last century, opioids related to or from opium came into existence, including their related problems. It remains unclear how these opioids affected SCD. The primary objective of this manuscript was not to have a detailed review about sickle cell disease and its related basic and clinical aspects. Most of these aspects have been described before, including by the NHLBI (National Heart, Lung and Blood Institute), American Society of Hematology and others. In the following paragraph, some of these aspects are described.

Management of sickle pain includes pharmacologic and nonpharmacological approaches. The former includes opioids, Nonsteroidal anti-inflammatory drugs (NSAIDs), acetaminophen, anesthetics, sodium channel blockers, gabapentinoids (Neurontin and Gabapentin), tramadol, tapentadol, cannabinoids, venom-derived compounds (Ziconotide) and a few others. Among these, opioids emerge as the compounds associated with many superlatives. Thus, opioids have the longest history of use; they are most potent, most commonly in use, misuse and abuse, associated with most serious side effects and are the most controversial. For patients with SCD, opioids are the most desirable analgesics for severe pain. This review will address the major aspects of opioids commonly used in the treatment of sickle cell pain. These span from ancient times to current controversies. The following definitions set the stage for further elaboration on the subject.

**Figure 1:**
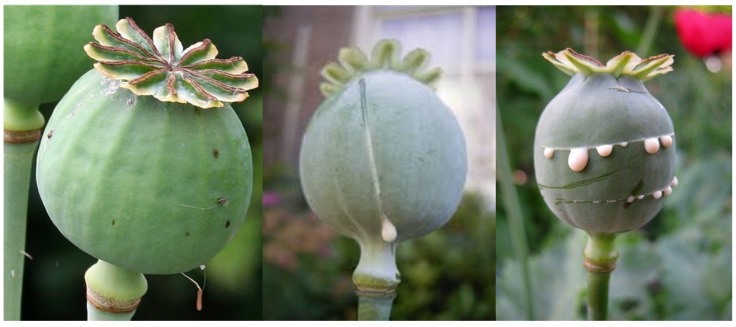
Opium poppy. Public domain photograph.

**Figure 2:**
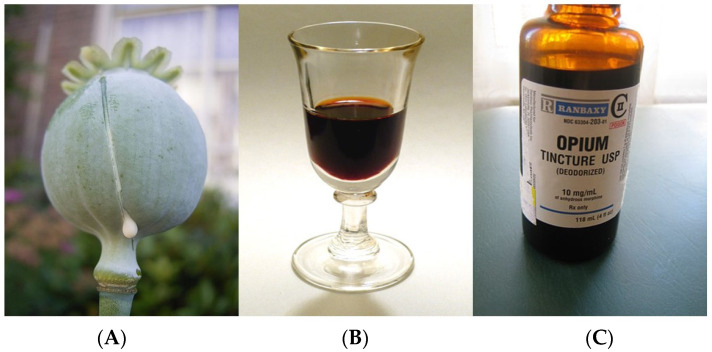
(**A**) Opium poppy seed pod exuding latex from a cut is mixed with alcohol (**B**) to make Laudanum (**C**) known as Opium Tincture. Photographs are public domain.

The word “opioid” refers to all analgesic compounds that possess morphine-like properties, whether they are naturally occurring, semisynthetic, synthetic, endogenous or exogenous. The word “opiate” refers to naturally occurring alkaloids, such as morphine, codeine, papaverine, thebaine and α-narcotine, derived from opium. “Opium” is the dried, powdered mixture of 20 alkaloids obtained from the unripe capsules of opium poppy seeds (*Papaver somniferum*) shown in Figure 1 [4,5,6]. Other poppy plants, such as the California poppy (*Eschscholzia californica*), contain small amounts of opium. As a matter of fact, California poppy is legally available as a fluid or seeds (Figure 2) over the counter and in food stores. The amount of opium in these is negligible but strong enough to be detected in urine drug screening tests.

The word “narcotic” is derived from the Greek “narkoun”, meaning “to benumb”, and “narki”, meaning “numbness” or “stupor” [6]. As a noun, narcotic means (1) any drug that induces stupor, (2) a person addicted to narcotics or (3) anything that causes stupor [6]. It is important to note that “opioids” and “narcotics” are not synonymous. In common usage, narcotic connotes addiction, drug-seeking behavior and association with abused substances. It is advisable not to use this term in a pharmacologic context.

## 2. Historic Milestones: From Opium to Opioids

### 2.1. Mythology

In Greek mythology, Hypnos (Somnus in Roman mythology) was the god of sleep. He slept in a cave in Hades with poppies growing at the entrance of the cave along with other hypnotic plants. The words hypnosis, hypnotic and insomnia are derived from their Greek and Roman names. Morpheus (god of dreams) was the eldest son of Hypnos. He had wings to reach people who needed help in their dreams and to communicate the messages of the gods to humans during sleep. He could take the form of any human in dreams. Each sibling of Morpheus was a God of different kinds of dreams such as nightmares and unrealistic dreams etc., including Phobetor (god of nightmares) and Phantasus (god of imagination and fantasy). Ironically, these gods, in a way, depict or represent some of the side-effects of opioids, including drowsiness, vivid dreams and fantasy dreams [6].

The Algea (Lupe, Ania and Achus) were the spirits who represent pain and suffering. The origin of the term analgesia is from a compound word of “an” (no) and “algia” (pain) [6].

### 2.2. Global Use of Opium

**Figure 3:**
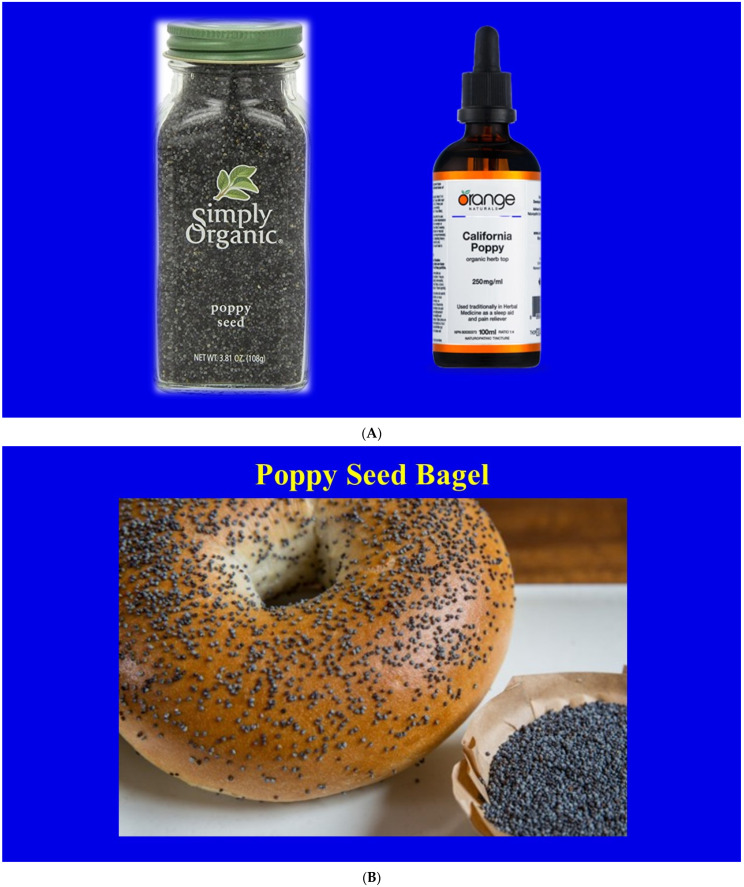
California poppy seeds have a culinary function and may be used as supplement (**A**) California poppy seeds for baking and herbal use. Photographs are public domain. (**B**) California poppy seed bagel. Photograph is public domain.

The Sumerians are credited for their use of opium in 3400 B.C. [7]. From Sumer, opium spread to Assyria and later to Egypt [7]. The ancient Greek poem, the Odyssey, mentions how familiar the Egyptian and Greek royalty were with opium [8]. The Egyptians used it as a tonic for children with abdominal pain [7]. Dioscorides, a first-century Greek physician-botanist, described how to collect opium from the unripe poppy seed in detail [7]. With time, the knowledge of the effects of opium spread globally from China and India to Europe and eventually to the Americas [7]. In the 16th century, Paracelsus (1493–1541), a Swiss-German physician-philosopher-alchemist-botanist, popularized the use of opium as a general analgesic [7]. He developed alcoholic solutions of opium which he called “Laudanum” (from Latin Laudare which means to praise) [7]. Laudanum (Figure 3) is basically a tincture of opium containing about 10% of opium powder [7]. Through the 19th century, Laudanum and powdered opium were used globally for both medical and recreational purposes. Medically, it was used as an analgesic and as a cough suppressant [7].

### 2.3. The Opium Wars

**Figure 4:**
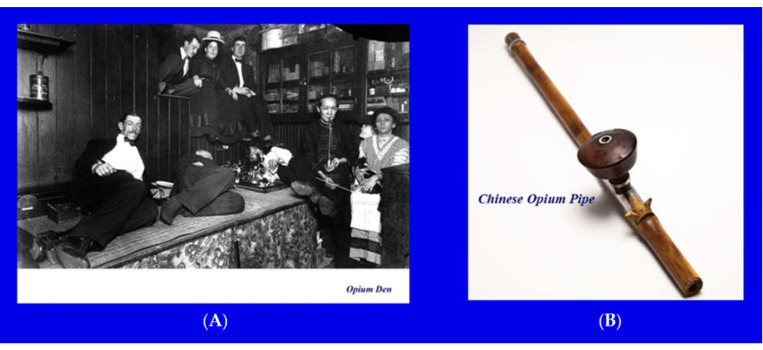
Opium den and pipe. (**A**) Opium den and (**B**) pipe. Photographs are public domain.

Recreational use of opium created a robust international opium trade. The opium trade, however, led to conflicts between China and certain European countries, mostly Great Britain [7]. The source of the conflict goes back to 1644, when the Chinese emperor, Tsung Chen, banned the popular habit of smoking tobacco. Consequently, the Chinese people turned to opium gradually as a desirable alternative [7]. By the 19 century, over 12 million Chinese were addicted to opium, and opium dens (Figure 4), as sites to buy and sell opium, spread over the country [7]. Foreign merchants, especially the British, found the soaring demand for opium in China a lucrative and profitable market [7]. By the 1830s, the British East India Company supplied more than one million pounds of Indian opium annually to China [7]. The Chinese authorities closed certain Chinese ports to the British in an effort to stop the opium trade, which crippled the Chinese society [7]. This led to the first (1836–1842) and second (1856–1860) opium wars between Great Britain and the Qing dynasty of China [7]. The outcome of these wars was the defeat of China, which signed a treaty to cede Hong Kong to Great Britain (which remained a territory up to 1999) and to give British merchants free trading rights [7].

### 2.4. Pharmacologic Discoveries and the Opioid Epidemic

After the opium wars were over, another major milestone emerged in the history of opioids. This was the discovery that the acetylation of a compound may result in a new compound more potent than its parent compound [7]. Thus, in 1874, the British scientist C. R. Alder Wright acetylated morphine to produce diacetylmorphine, also known as heroin [7].

By the early 20th century, laudanum was sold without a prescription and was a constituent of many medicines [7]. Currently, laudanum is recognized as addictive and is strictly regulated throughout most of the world. It is more commonly referred to as a tincture of opium and is available as a prescription drug. The United States Uniform Controlled Substances Act, for example, lists it as a Schedule II drug. Heroin, on the other hand, was introduced for medical use in 1898, but by 1903, its abuse soared in the US. Heroin use was made illegal by federal law in 1924.

Throughout the 20th century and early 21st century, there has been a beehive activity in the production of semi-synthetic, synthetic, extended release and long-acting opioids. The mechanism of the action of opioids was clarified and the use of opioids for diseases associated with pain soared. However, the introduction of the concept of pain as the fifth vital sign and the heavy marketing of certain opioids seem to be some of the reasons that led to increased utilization of opioids that was, unfortunately, associated with the abuse and misuse of opioids and the current opioid epidemic in the United States.

## 3. Classification of Opioids

Opioid analgesics are classified in several ways [9,10,11,12,13,14,15,16]. One classification divides them according to their source as naturally occurring, semisynthetic and synthetic, as shown in Table 1.

Physiologically, opioids are classified according to their binding and interaction with specific receptors. Specifically, opioids are classified as agonists, partial agonists, mixed agonist-antagonists and antagonists, as shown in Table 2 [17]. Another classification divides opioid arbitrarily into weak and strong groups according to their potency and efficacy. Weak opioids are available in combination with nonopioid analgesics, such as acetaminophen or aspirin. To provide additive antipyretic and anti-inflammatory effects, they limit the amount that can be administered, because a daily maximum of 4000 mg acetaminophen, an amount contained in approximately 12 tablets of Percocet, is recommended to avoid hepatotoxicity.

## 4. Pharmacodynamics of Opioids

Pharmacodynamics is the branch of pharmacology that studies the mechanisms of the action of drugs and their biochemical and physiologic effects [19]. It is often referred to as the branch of pharmacology that studies the effects of the drug on the body [19]. Most drugs exert their effects via interactions with tissue receptors to which they are bound, and hence, trigger a series of biochemical and physiologic cellular events that culminate in a response characteristic of the drug in question [19]. This sequence of events can be illustrated schematically as follows:

Drug + receptor → drug/receptor complex → response [17].

### 4.1. Mechanism of Action of Opioids

Opioid receptors are G protein-coupled receptors with exogenous and endogenous opioids as ligands [20]. Opioid receptors were first discovered in the 1970s [21]. There are three major types of opioid receptors, namely, μ, κ and δ, and eight subtypes of these receptors, termed μ1, μ2, μ3, κ1, κ2, κ3, δ1 and δ2. Opioids interact with various receptor subtypes. Adverse effects depend on the degree of binding to receptors. To date, no known opioid analgesic selectively activates μ1 receptors without concomitant activation of the other μ receptors. Such a drug would be ideal to produce analgesia without sedation. Opioid analgesics that bind more to μ2 receptors, which mediate sedation, and less to μ1 receptors, which mediate analgesia, might produce excessive sedation without adequate analgesia, that is, a sleepy patient who complains of severe pain whenever awakened. This situation could create logistical problems in managing painful VOCs [17]. All opioid agonists bind primarily to µ receptors and less actively to δ receptors. Buprenorphine is a partial agonist of µ receptors. Naloxone and Naltrexone are antagonists to all receptors.

**Figure 5:**
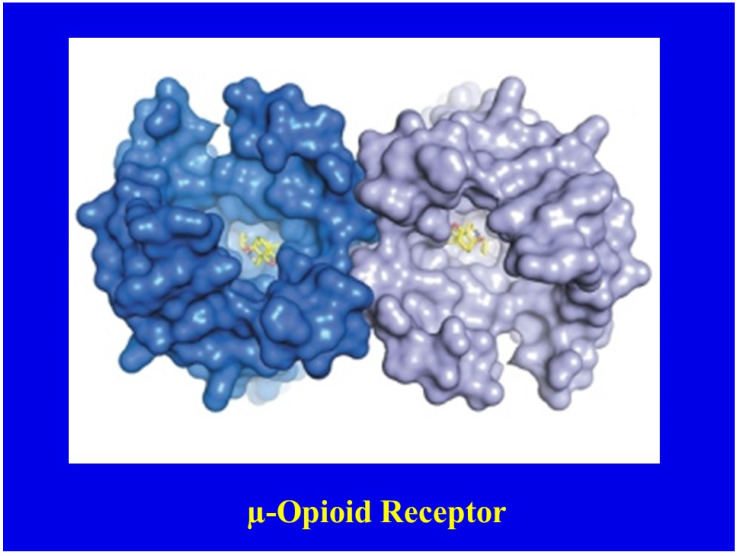
Helical structure of the μ-opioid receptor. Reproduced with permission from Manglik, A.; *Nature*; published by Nature Publishing Group, 2012 [24].

Elegant studies [22,23,24,25,26] have revealed a helical structure of the opioid receptors, which forms pockets in which the corresponding ligand (opioid) fits snugly (Figure 5). Receptors mediate two major functions, chemical recognition and physiologic action. Recognition is highly specific, such that only L-isomers of opioids exert analgesic activity [27]. The binding affinity, or strength with which a drug binds to its receptor, varies considerably among opioids [28]. For example, fentanyl has a higher binding affinity than morphine [27]. The binding affinities of opioids appear to correlate well with their analgesic potencies [29]. Physiologically, by binding to receptors, opioids initiate a series of biochemical events, including the activation of G proteins, inhibition of adenylate cyclase and extrusion of potassium ions, resulting in hyperpolarization of cell membranes [30,31,32]; this delays or prevents the transmission of painful stimuli.

**Figure 6:**
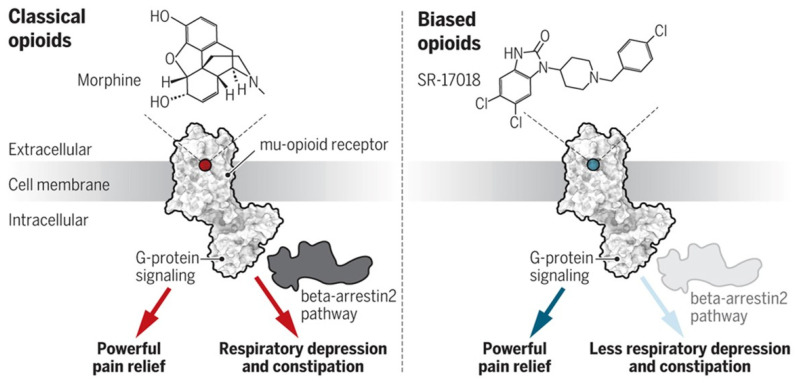
Classical Opioids vs. Biased Opioids. Classical opioids transmit both the beneficial and the harmful side effects. Biased opioids transmit only the beneficial effects. Reproduced with permission from Servick, K., *Science*; published by American Association for the Advancement of Science, 2020 [33].

The problem with the µ opioid receptors is that they transmit both the analgesic effects and the side effects, especially the respiratory suppression. Attempts have been made to design opioids that are “biased” toward activating painkilling signals only while leaving the undesired side effects alone (Figure 6) [33]. To date, this approach has been used in mouse models with questionable efficacy. Research in this line of studies is ongoing.

### 4.2. Side Effects of Opioids

Major side effects of opioids are listed in Table 3 [9,12,13,14,15,16,34]. Some side effects, such as anxiety relief, euphoria and sedation, are desirable in managing acute sickle pain. Neurologic side effects include euphoria, drowsiness, mental confusion and apathy. Nausea and vomiting ensue from direct stimulation of medullary emetic chemoreceptors.

The chronic use of opioids causes hypogonadism, due to central suppression of the hypothalamic secretion of gonadotropin-releasing hormone. Symptoms of opioid-induced hypogonadism include menstrual irregularities and galactorrhea in women, impotence in men, loss of libido, infertility, fatigue, anxiety, loss of muscle strength and mass, osteoporosis and compression fractures [36,37,38,39].

Dental Complications of Sickle Cell Disease include dental caries, dental erosions, infractions, hypodontia, malocclusions, pulp necrosis, abnormal trabecular spacing and infection [40].

Pulmonary effects include diminished tidal volume followed by depressed responses of the respiratory center to carbon dioxide. Cardiovascular effects include depressed responsiveness of α-adrenergic receptors, causing peripheral vasodilation, reduced peripheral resistance and inhibited baroreceptors, which may result in orthostatic hypotension. Gastrointestinal effects include inhibition of peristalsis, which may cause constipation and spasm of the sphincter of Oddi. Urinary tract manifestations are primarily urinary retention due to enhanced bladder sphincter tone.

The excessive use of opioid analgesics may precipitate acute chest syndrome due to their depressive effect on respiration. Recommendations to use nonsteroidal anti-inflammatory drugs (NSAIDs) should be considered carefully [17]. Opioids have less systemic side effects, and careful monitoring of their use ensures their safety. They should be discontinued if the respiratory rate is ≤10 breaths per minute, and their adverse effects can be quickly reversed with opioid antagonists. On the other hand, NSAIDs have more systemic side effects that may not be readily obvious. For example, NSAIDs decrease the levels of prostaglandins and prostacyclin, prostanoids that are essential in modulating the vascular tone of smooth muscle and renal blood flow. Thus, NSAIDs may worsen the clinical picture of ACS (Acute chest syndrome) due to their vasoconstrictive effects and bronchospasm; NSAIDs are contraindicated in asthma for the same reasons. NSAIDs use was associated with an increased risk of asthma exacerbation. In general, NSAID-induced bronchospasm develops within 30 to 180 min (sometimes up to 24 h) after drug ingestion, possibly precipitating the asthma exacerbation [41,42].

Opioids have abuse potential; psychologic dependence or addiction, physical tolerance and physical dependence may develop with repeated use. Other complications include skin rash, itching and CNS (Central nervous system) hyperirritability, with toxic doses manifesting in multifocal myoclonus and seizures. Meperidine is most notorious for the latter complication; repetitive dosing results in the accumulation of the active metabolite normeperidine, which produces hyperirritability, including seizures. However, seizures can occur with toxic doses of most opioid analgesics [10,43].

Treatment of severe opioid withdrawal includes methadone plus clonidine either orally (0.1–0.2 mg every 4–6 hours prn (Pro re nata)) or by using transdermal clonidine patch 0.1 mg daily. Other drugs that may be used to treat withdrawal include buprenorphine plus naloxone orally [44,45]. The FDA approved oral lofexidine to treat the symptoms of withdrawal [46]. Lofexidine is a structural analog of clonidine. Clinical trials comparing the two medications showed comparable efficacy, though the severity of adverse events was less than those with clonidine. This decreased risk for adverse effects could potentially make lofexidine a safer option for detoxification [47,48,49].

Tolerance is defined as reduced potency of the analgesic effect of an opioid after repeated administration or the need for higher doses to maintain the same result. It shifts the dose-response curve to the right, indicating that a higher dose of opioids is required to maintain the same level of analgesia [50]. The binding of an opioid to its receptor generates a series of reactions that could culminate in tolerance [51]. Studies in mice have shown that tolerance to morphine seems to be modulated by the gut-microbiome-central nervous system interactions [52,53,54].

Management of opioid tolerance entails the use of N-Methyl-D-Aspartate (NMDA) inhibitors. The NMDA channel is a complex structure [55]. It is both a receptor and a calcium-gated channel [56,57]. Therapeutic inhibitors of NMDR include ketamine, clonidine, Lidocaine, dextromethorphan, nitrous oxide, zinc and methadone [51,58,59]. More recently, rosuvastatin, B vitamins and inhibition of platelet-derived growth factor-β (PDGFR-β) have been shown to attenuate or eliminate the development of tolerance to morphine in rats and mice [60,61,62,63].

Dependence, also termed physical dependence, is a common and natural result of the body growing used to a drug or medication, particularly an opioid such as morphine. If the drug were suddenly stopped, the patient would undergo physical problems associated with withdrawal (also termed abstinence syndrome). It is easily avoided by reducing the dose of the drug gradually. It is distinct from addiction and tolerance.

Opioid-induced hyperalgesia (OIH) is defined as increased sensitivity to pain stimuli (hyperalgesia) and pain caused by ordinarily nonpainful stimuli (referred to as allodynia). Typically, hyperalgesia is noted in parts of the body different from the site of the original pain complaint, and the descriptors of the pain change with some similarity to certain aspects of neuropathic pain, such as burning sensation. Unlike tolerance, OIH worsens with higher doses of opioids [64,65,66].

The pathophysiology of OIH is not well understood. A proposed mechanism is the activation of the NMDA receptor [64,67]. This activation results in calcium influx, which in turn enhances the excitability of neurons, which facilitates further transmission of painful stimuli [59].

Management of OIH involves weaning from opioids, opioid rotation and the use of NMDA inhibitors such as methadone, clonidine, Lidocaine or ketamine, as needed. Weaning and rotation are usually done together.

Pseudoaddiction is a syndrome of behavior and attitudes that emerges in patients who are not being provided with adequate analgesics.

Physical dependence, addiction, tolerance and hyperalgesia may become confused by health care providers and used interchangeably because these conditions may occur together in the active drug abuser. Drug-seeking behavior is rare in patients with SCD taking opioids under medical supervision, and the incidence of iatrogenic addiction is low [68,69].

## 5. Pharmacokinetics of Opioids

Pharmacokinetics is the branch of pharmacology that studies the factors affecting drug movement in the body. Unlike pharmacodynamics, the focus of pharmacokinetics is the effects of the body on the drug including absorption, distribution, metabolism and excretion [19].

### 5.1. Metabolism of Opioids

The metabolism of opioids includes two major phases shown in Table 4 [17,70]. Phase I involves the cytochromes P450 (CYPs) enzymes, whereas glucoronidation is the major metabolic pathway in phase II metabolism.

The CYP superfamily is a large and diverse group of enzymes that catalyze the oxidation of organic substrates, including metabolic intermediates such as lipids and steroid hormones, drugs and toxic chemicals. Human CYP consists of 21 currently described families and 20 subfamilies coded by 57 genes. The CYP isoforms 1, 2 and 3 are responsible for the majority of hepatic drug metabolism [17]. Of these, CYP2C9 and CYP2D6 are involved in the metabolism of several drugs used for pain control including opioid and nonopioid analgesics. Phase I metabolism of opioids involves primarily the CYP3A4 and CYP2D6 enzymes. The CYP3A4 enzyme metabolizes more than 50% of all drugs; consequently, opioids metabolized by this enzyme have a high risk of drug–drug interactions [17,70]. Thus, medications used in addition to opioids may enhance or inhibit the metabolism of the opioid in question.

A major issue in the pharmacogenomics of the CYP 450 system is that the enzymes may be deleted, mutated, duplicated or even triplicated. The genotypes of the CYP 450 are categorized into phenotypes based on the activity of the variant enzymes. Ultrarapid metabolizers (UMs) have greater-than-normal activity due to duplication or triplication, of active alleles [71,72], extensive metabolizers (EMs) have normal enzyme activity, intermediate metabolizers (IMs) have decreased enzyme activity and poor metabolizers (PMs) have absent or little enzyme activity. Thus, the metabolic activity of a certain enzyme could be normal (function 100%), intermediate (function 50%), poor (function 0–10%) or ultrarapid (function >100%). The clinical effects of CYP2D6 allelic variants are best demonstrated with codeine administration. Patients who are poor opioid metabolizers experience reduced efficacy with codeine because of their limited ability to metabolize codeine to its active metabolite morphine. In contrast, patients who are ultrarapid opioid metabolizers may experience increased opioid effects with a usual dose of codeine because their rapid metabolism generates a high concentration of morphine [17].

Phase II metabolism involves morphine, where it mostly undergoes glucuronidation. Moreover, morphine metabolism is subject to genetic variability due to allelic variants in a number of genes. Variations in these genes may explain the variability in efficacy and potency of morphine in patients with SCD and in patients with other pain syndromes. Most important among these include the uridine diphosphate glucuronyltransferase enzyme (UGT2B7, located on chromosome 4q13), the μ-opioid receptor 1 gene (OPRM1; located on chromosome 6q24–q25) and catechol-O-methyltransferase (COMT; located on chromosome 22q11.21). The presence of the UGT2B7 promoter variant—840 G→A is reported to decrease morphine glucuronidation in patients with SCD [17]. The frequency of this allele among the 20 patients with SCD studied was 70%. In comparison, the UGT2B7 promoter variant—79 G→A has no effect on morphine glucuronidation.

The OPRM1 gene encodes a μ-opioid receptor belonging to the G protein-coupled membrane receptor family [17]. An allelic variant of OPRM1, 118 A→G, has been implicated in lowering the potency of morphine and the level of M6G in patients with the A/G genotype. Patients homozygous for the G allele (GG) required larger doses of morphine to achieve pain relief [17].

In addition, the pharmacokinetics of morphine in patients with SCD appears to differ from that in patients with other pain syndromes. Studies of children with SCD treated with intravenous morphine during painful VOC found increased clearance of morphine, particularly in prepubertal children [17], which was significantly greater than that in studies conducted in children with postoperative pain or cancer pain [17]. Similarly, increased clearance of morphine was reported in young adults (≥18 years) with SCD in a steady state in the absence of painful VOC [17].

### 5.2. Drug–Drug Interactions

Patients with sickle cell pain often receive a combination of various drugs, including opioid analgesics, nonsteroidal anti-inflammatory drugs (NSAIDs), adjuvant analgesics and antibiotics.

Such drugs may interact, and the response elicited may be equal to, greater than or less than the sum of the effects of the individual compounds. Synergism, potentiation, additive effect and antagonism are some of the common terms that describe the pattern of drug–drug interactions [17].

Coadministration of other drugs with opioid analgesics requires skillful selection and monitoring, especially when a centrally acting drug is used. The sedative effects of an opioid may accentuate that of other agents such as antidepressants, neuroleptics and anxiolytics. Drugs with anticholinergic effects may worsen constipation caused by opioids [43,73,74].

Generally, the effects of morphine can be antagonized by acidifying agents and potentiated by alkalinizing agents. The concomitant use of anticholinergics with opioids, including morphine, may result in an increased risk of severe constipation and urinary retention.

Central nervous system depressants, such as other opioids, alcohol, anesthetics, antihistamines, barbiturates, β-adrenergic blocking agents, chloral hydrate, glutethimide, hypnotics, monoamine oxidase inhibitors, phenothiazines, pyrazolidone, sedatives, skeletal muscle relaxants and tricyclic antidepressants, can enhance the depressant effects of morphine. Concurrent use may result in potentiation of CNS depression, and death may occur. If used concurrently with CNS depressants, dosage adjustment may be required [43,73,74].

Amphetamines potentiate the analgesic effect of opioids. Opioids can increase the anticoagulant activity of warfarin and other anticoagulants.

### 5.3. The Opioid Epidemic and the Use of Opioids in SCD

The advent of the opioid epidemic had a negative effect on the management of sickle cell pain. Some providers found the opioid epidemic a justification to minimize the use of opioids for sickle cell pain. Alternatives to opioids for sickle cell pain are not available yet. The status of cannabinoids is unsettled and Kratom failed as replacements to opioids [75,76]. The systemic side effects of NSAIDs in adults such as renal failure, cardiovascular compromise and gastrointestinal bleeding are worse than the systemic side effects of opioids [17]. The major cause of death in SCD is not opioids [77] but the complications of the disease such as infection, acute chest syndrome, renal failure, stroke and multiorgan failure. In addition, the use of opioids by patients with SCD remained constant over the years [78].

## 6. Conclusions

Currently, there is a plethora of opioid preparations on the market, especially in the USA. Opioids come in different formulations, shapes, sizes, doses, tablets, capsules, fluids, patches or inhalers. They are among the best pain relievers and, unfortunately, the worst drugs that are amenable for misuse associated with high mortality. Thus, the problem with opioids is not the drugs themselves but the way they are used. Fortunately, opioids seem to be used wisely by the majority of the providers who treat patients with SCD. Mortality in patients with SCD is primarily due to the complications of the disease itself and not the opioids. Issues that are not finalized in SCD pertain to determining the best opioid(s) to manage acute, chronic, neuropathic pain as well as other types of pain. The most commonly used opioid in SCD changed with time. In the late 20th century, meperidine was used most commonly. Later, this was replaced by morphine, hydromorphone and fentanyl. Currently, buprenorphine seems to be on the rise to be the best to treat acute and chronic sickle cell pain, and in combination with Naloxone, it is the best to prevent and treat addiction and withdrawal.

## Figures and Tables

**Table 1:** Classification of opioids according to source.

**Natural** Morphine, Codeine, Papaverine, Thebaine
**Semi-synthetic** Heroin, Hydrocodone, Hydromorphone, Oxycodone, Oxymorphone, Naloxone, Naltrexone, Nalmefene Nalbuphine, Buprenorphine, Butorphanol
**Synthetic** Meperidine, Fentanyl, Methadone, Levorphanol
**Endogenous** Enkaphelin and Endorphin

**Table 2:** Classification of opioids according to chemical structure and function.

**Agonists** Naturally occurring (opium alkaloids) Codeine Morphine Papaverine Semisynthetic opioids Hydrocodone (Hycodan, Vicodin, Lortab, Tussionex) Oxycodone (Percocet, Percodan, Roxicet, Roxicodone, Tylox, Oxycontin) Hydromorphone (Dilaudid) Oxymorphone (Numorphan **, Opana, Opana ER) Synthetic opioids Morphinans Levorphanol (Levo-Dromoran) Phenylpiperidines Meperidine (Demerol, Pethidine) Alfentanil and Remifentanil Fentanyl (Sublimaze, Durgesic, Actiq, Fentora, Lazanda, Onsolis) Sufentanil Diphenylheptanes Methadone (Dolophine) Propoxyphene HCl (Darvon, Darvocet, Wygesic) * Propoxyphene Napsylate (Darvon N) * Partial agonists Buprenorphine (Buprenex, Subutex, Butrans, Suboxone) Dezocine (Dalgan) ^†^ **Mixed agonists-antagonists** Pentazocine (Talwin, Talwin NX) Nalbuphine (Nubain **) Butorphanol (Stadol ^††^) **Other** Tapentadol (Nucynta)

* Propoxyphene and all combination drugs containing it were withdrawn by the FDA in 2010, and other countries are doing the same. Readers are advised to check its status in their countries. ^†^ Dezocine is not available in the United States and Canada. However, in China, it is used after surgery. ** Brand has been discontinued in the US. ^††^ Metered spray and nasal forms of this drug have been discontinued in the US [17]. A relatively recent classification is to divide opioids into short-acting and Extended Release and Long-Acting (ER/LA) [18]. Short-acting opioids are usually used for acute pain and ER/LA opioids for chronic pain [18]. In order to shift from one opioid to another, equianalgesic potency has to be taken into consideration. One method of equianalgesic dosing is shown in Table S1 [17].

**Table 3:** Opioid risks.

**1.** **Mild/Moderate Side Effects:** Sedation Confusion Nausea Dizziness Constipation
**2.** **Serious Medical Side Effects:** Gonadal suppression Respiratory suppression Sleep apnea Dental complications
**3.** **Serious Neurological and Behavioral Side Effects:** Physical dependence Withdrawal Tolerance Hyperalgesia Addiction Pseudo addiction Abuse, misuse, diversion

Adapted from Ballas, S.K. [35].

**Table 4:** Metabolic pathways of commonly used opioids in sickle cell disease.

Opioid	Phase I CYP450	Phase II Glucuronidation	Active Metabolite	Inactive Metabolite	Non-Opioid Active Metabolite
Morphine	None	Yes	Hydromorphone	Normorphine	M6G, M3G
Hydromorphone	None	Yes	None	Minor Metabolites	HM3G
Oxymorphone	None	Yes	None	Oxy3G	6-OH-Oxymorphone
Codeine	CYP2D6	None	Morphine, Hydrocodone	Norcodeine	None
Hydrocodone	CYP2D6, 3A4	None	Hydromorphone	Norhydrocodone	None
Oxycodone	CYP2D6, 3A4	None	Oxymorphone	None	Noroxycodone
Fentanyl	CYP3A4	None	None	Norfentanyl	None
Methadone	CYP2D6, 3A4, 2C8, 2C9, 2C19,2B6, 1A2	None	None	2-C2H5-5-CH3-3,3diphenypyrrolidine	None
Tramadol	CYP2D6, 3A4, 2B6	None	None	Nortramadol	O-desmethyl-tramadol

M6G = Morphine-6-glucuronide; M3G = Morphine-3-glucuronide; HM3G = Hydromorphone-3-glucuronide; Oxy3G = Oxymorphone-3-glucuronide.

## Supplementary Materials

The following are available online at https://www.mdpi.com/2077-0383/10/3/438/s1, Table S1: Equianalgesic dosing equivalents of opioids in opioid-naïve patients.
