# Supplementary material for: Opioids and Sickle Cell Disease: From Opium to the Opioid Epidemic
Source: J Clin Med. 2021 Jan 23;10(3):438. doi: 10.3390/jcm10030438 (PMC7865837; doi:10.3390/jcm10030438)
Supplement: Supplementary file 1 [file jcm-10-00438-s001.pdf]

Table 1. S. Equianalgesic dosing equivalents of opioids in opioid naïve patients.

Drug	Potency i.v./p.o.	Equianalgesic Doses i.v. (mg)	p.o. (mg)
Agonists			
Morphine	3	10	30*
Hydromorphone	5	1.5	7.5†
Meperidine	4	75	300
Levorphanol	2	2	4
Fentanyl	NA	0.1	NA
Sufentanil	NA	0.02	NA
Methadone	2	5	10**
Codeine	1.5	120-130	200
Oxycodone (p.o.)	NA	NA	20
Propoxyphene (p.o.) ††	NA	NA	130
Oxymorphone	10	1	10†
Hydrocodone	NA	NA	30
Partial agonist			
Buprenorphine	NA	0.3-0.4	NA
Dezocine◊	NA	10	NA
Mixed agonist-antagonists			
Pentazocine	3	60	180
Nalbuphine	NA	10	NA
Butorphanol	NA	2	NA

* 60mg for single dose.

†Hydromorphone rectal is 3mg and Oxymorphone rectal is 10mg.

†† Propoxyphene and all combination drugs containing it were withdrawn by the FDA in 2010 and other countries are doing the same. Readers are advised to check its status in their countries.

◊ Dezocine is not available in the United States and Canada. However, in China, it is used after surgery.

Adapted from Ballas, S.K. *Sickle Cell Pain, 2nd Edition*; International Association for the Study of Pain: Washington DC, 2014.
